# Development of Multiplex qPCR Method for Accurate Detection of Enzyme-Producing Psychrotrophic Bacteria

**DOI:** 10.3390/foods14111975

**Published:** 2025-06-03

**Authors:** Kidane Yalew, Shuwen Zhang, Solomon Gebreyowhans, Ning Xie, Yunna Wang, Jiaping Lv, Xu Li, Xiaoyang Pang

**Affiliations:** 1 Key Laboratory of Agro-products Quality and Safety Control in Storage and Transport Process, Ministry of Agriculture and Rural Affairs, Institution of Food Science and Technology, Chinese Academy of Agricultural Sciences, Beijing 100193, China; 2018y90100121@caas.cn (K.Y.); zswmaster@163.com (S.Z.); xiening@caas.cn (N.X.); wang_yn92@163.com (Y.W.); kjdairy@126.com (J.L.); 2 Department of Vet. Public Health and Food Safety, College of Veterinary Science, Mekelle University, Mekelle 0231, Ethiopia; 3 Tigray Agricultural Research Institution, Mekelle 0492, Ethiopia; sgg1976bd@gmail.com

**Keywords:** hydrolytic enzymes, microbial detection, multiplex qPCR, psychrotrophic bacteria, raw milk

## Abstract

Microbial detection in milk is crucial for food safety and quality, as beneficial and harmful microorganisms can affect consumer health and dairy product integrity. Identifying and quantifying these microorganisms helps prevent contamination and spoilage. The study employs advanced molecular techniques to detect and quantify the genomic DNA for the target hydrolytic enzyme coding genes *lipA* and *aprX* based on the multi-align sequence conserved region, specific primer pair, and hydrolysis probes designed using the singleplex qPCR and multiplex qPCR. Cultured isolates and artificially contaminated sterilized ultra-high-temperature (UHT) milk were analyzed for their specificity, cross-reactivity, and sensitivity. The finding indicated that strains with *lipA* and *aprX* genes were amplified while the other strains were not amplified. This indicated that the designed primer pairs/probes were very specific to the target gene of interest. The specificity of each design primer pair was checked using SYBR Green qPCR using 16 different isolate strains from the milk sample. The quantification specificity of each strain target gene was deemed to be with a mean Ct value for positive pseudomonas strain > 16.98 ± 1.76 (*p* < 0.0001), non-pseudomonas positive strain ≥ 27.47 ± 1.25 (*p* < 0.0001), no Ct for the negative control and molecular grade water. The sensitivity limit of detection (LOD) analyzed based on culture broth and milk sample was >10^5^ and >10^4^ in PCR amplification while it was >10^4^ and >10^3^ in real-time qPCR, respectively. At the same time, the correlation regression coefficient of the standard curve based on the pure culture cell DNA as the DNA concentration serially diluted (20 ng/µL to 0.0002 ng/µL) was obtained in multiplex without interference and cross-reactivity, yielding R^2^ ≥ 0.9908 slope (−3.2591) and intercepting with a value of 37, where the efficiency reached the level of 95–102% sensitivity reached up to 0.0002 ng/µL concentration of DNA, and sensitivity of microbial load was up to 1.2 × 10^2^ CFU/mL. Therefore, multiplex TaqMan qPCR simultaneous amplification was considered the best method developed for the detection of the *lipA* and *aprX* genes in a single tube. This will result in developing future simultaneous (three- to four-gene) detection of spoilage psychrotrophic bacteria in raw milk.

## 1. Introduction

Microbial detection in milk is crucial for ensuring both food safety and quality [1,2,3]. The presence of microorganisms in milk, including both beneficial and harmful species, can affect consumer health and the integrity of dairy products [4]. Ensuring the microbiological quality of milk involves detecting and quantifying these microorganisms to prevent contamination and spoilage [5].

Detection of psychrotrophic bacteria is critical for assessing the quality of raw and pasteurized milk, as their presence can significantly affect shelf life and spoilage [6,7,8]. These bacteria are significant in various industries for their capability of extracellular enzyme production that can resist heat, including lipases and proteases post-pasteurization, which are essential for the hydrolysis of fats and proteins, leading to spoilage and quality degradation. Various genera, such as *Pseudomonas*, *Acinetobacter*, and *Lactobacillus*, have been documented as enzyme producers at low temperatures and are often associated with shelf life reduction through continuous enzymatic activity during storage and economic loss leading to product recalls, consumer complaints, and brand reputation damage. Process disruption in cheese production can also occur sue to uncontrolled proteolysis crud formation and ageing kinetics, while lipolysis can affect flavor profile defects and other quality issues in dairy products [9,10,11].

Quantitative real-time PCR (qPCR) and multiplex qPCR have emerged as rapid, sensitive, and specific tools for identifying and quantifying spoilage microbes, enabling proactive quality control in the dairy supply chain superior to traditional culture-based detection methods which are time-consuming (24–72 h) and often fail to detect low-abundance or non-culturable microbes [12,13,14]. The molecular identification of spoilage psychrotrophic bacteria has gained prominence as it allows precise detection and variation of species present in milk and dairy products [14,15,16], packed meat [17,18], seafood [19], and yogurt [13].

According to recent research, qPCR and multiplex qPCR are extremely sensitive methods for identifying pathogen bacteria and food spoilage, with detection limits that vary from 10 to 10^3^ CFU/g depending on the target and food matrix. Using qPCR, food pathogens such as *Salmonella enterica* and *Escherichia coli O157:H7* were detected 10 to 10^2^ CFU/mL in contaminated foods following enrichment, and the limit of detection for the spoilage bacteria *Pseudomonas* spp., which is common in milk was 10^2^ CFU/mL. The sensitivity simultaneous multiplex qPCR of pathogens for *Listeria monocytogenes*, *Salmonella* spp., and *Escherichia coli* O157:H7 in food achieved 10^2–^10^3^ CFU/g for each pathogen in spiked food samples and spoilage bacteria 10^3^ CFU/g *pseudomonas* spp. in seafood [20,21,22]. The future of microbial detection in milk is likely to see advancements in rapid testing technologies and automation. New methodologies, such as automated digital PCR (dPCR) systems for real-time detection of microorganisms to enhance the sensitivity and efficiency of microbial analysis in the dairy industry, have been developed [23,24].

Therefore, as consumer awareness around food safety increases, the demand for high-quality, microbiologically safe dairy products is expected to grow, driving continued innovation in simultaneous detection psychrotrophic bacteria producing hydrolytic enzyme in milk. It is vital, time-saving, cost-effective, and more specific than uniplex fashion. This will improve quality control and minimize economic losses in the dairy industry.

## 2. Methods and Materials

### 2.1. Bacteria Culture Growth

From the total of 33 isolate bacteria listed in Table S1, the selected group of bacteria for this research study are listed in Table 1. All of them were isolated from 15 chilled raw milk samples of 50 mL collected in a sterile glass and transported in ice to our laboratory from 5 different dairy farms in the Hebei province of China. Psychrotrophic bacteria species were cultivated under aerobic conditions on LB nutrient agar (Qingdao Bio-Tech Co., Ltd., Qingdao, China) incubated at 4 °C for 10 days. Cell counts were determined on LB plate count agar after incubation in 28 °C orbital shaker with 150 rpm for 24–48 h.

### 2.2. Screening of Target Gene

Genomic sequence of different bacterial species and *Pseudomonas* strains with a target gene fasta was downloaded from the NCBI gene bank using the keywords triacylglycerol lipase and *aprX* gene. Hundreds of strains were available, but only eight strains with *lipA* gene and seven with *aprX* gene, including the noble strain *P. fluorescens* and non-Pseudomonas lipase positive, were selected for this research. Then, the selected strain’s sequence fasta was aligned using the Ugene software (Version 1.29.0) [25] for multiple sequence alignment; the candidate target conserved region reference was used, with the noble strain *P. fluorescens* as a target.

### 2.3. Genomic DNA (gDNA)

Genomic DNA was extracted from a broth culture bacteria revived in LB broth (Qingdao Bio-Tech Co., Ltd., Qingdao, China) centrifuge using 3K15 Benchtop (Sigma, Ontario, CA, USA) at room temperature (RT) at 10,000 rpm for 1 min, while the milk sample was centrifuged on RT at 6000 rpm for 30 min to remove the upper layer of fat with the supernatant. gDNA from the residual sediment cell was extracted and eluted based on the protocol using a TIANGEN bacteria gDNA Kit^®^ (Tiangen Bio-Technology, Co., Ltd., Beijing, China). Eluted gDNA with a volume of 100 µL molecular-grade water (Biorigin, Beijing, China) was checked for purity, and its concentration ratio @260/280 was determined using the Nucleic Acid Micro spectrophotometer K5500 Plus (Xi’an Hub Biotechnology Co., Ltd., Xi’an, China); it was stored at −20 °C until use to prevent DNA lysis. The gDNA of pure culture and artificially contaminated milk samples were further diluted to a working concentration of 50 ng/µL and 20 ng/µL for the PCR and qPCR, respectively, to verify our estimated result quantification. The limit of detection was set based on the plate count CFU/µL.

### 2.4. Primer Pair and Hydrolysis Probe Designing

Different online applications and primer design software were used to design the intended primer pair amplification of the candidate target genes, such as Primer3, NCBI primer pick, SnapGene (version 6.0.2), Primer Premier 6, and Genescript (https://www.genscript.com/) [26]. The designed primer pairs were selected from different regions of the candidate target gene multi-align sequence conserved region, focusing on the triacylglycerol lipase for *lipA* and *aprX* for the protease enzyme produced targeting the noble strain *P. fluorescens.* The primer pair and hydrolysis probe design listed in Table S2, Table 2 were synthesized at the Beijing genomic institution (BGI, Beijing, China).

### 2.5. PCR and qPCR Condition Setup

#### 2.5.1. PCR Base

The PCR mix was prepared using the TianGen 2xTaq PCR Master Mix kit (Tiangen, Beijing, China); the mix reaction volume was 20 µL with 2xTaq PCR MasterMix 10 µL, 1 µL each primer (F&R), 7 µL molecular-grade water, and 1 µL templet DNA of 50 ng/µL on a PCR system Applied Biosystems 96 well (Veriti^TM^ Thermal Cycler, Milford, CT, USA) Thermo Fisher Scientific, program: 95 °C: 5 min, 30 cycles (95 °C: 30 s, 60 °C: 30 s, 72 °C:1 min). The final step was performed at 72 °C: 10 min. The mixture was verified on 2% agarose gel (Biowest, Madrid, Spain) electrophoresis (AGE) and visualized using 10,000× gene green nucleic acid dye (Tiangen, Beijing, China) for the visibility of clear band based on the DNA marker (Tiangen, Beijing, China) using a Gel-image scanner (Clinx^®^, Shanghai, China).

#### 2.5.2. TaqMan Probe Base

For amplification of single and multiplex qPCR, the total reaction volume was used as per the kit protocol 20 μL, with 10 μL of Super Real TaqMan mix (TianGen^®^,Beijing, China), with primer final optimized concentration of 200 nM, probe final optimized concentration of 150 nM, 2.5 μL template DNA of 20 ng/µL and molecular-grade water up to the final volume. ABI7500 real-time PCR 96 Systems (Applied Biosystems) thermal cycling was performed, with the following protocol: Step one—holding stage at 50 °C: 2 min, second holding stage 95 °C: 10 min, then a 40-cycle denaturation at 95 °C: 15 s, annealing optimized at 60 °C: 1 min, and extension at 72 °C:1 min. The qPCR assays were performed in triplicate, and water was used to ensure the absence of contamination as a no template (NT) and UHT milk DNA was used as negative control.

### 2.6. Primer Pair and Hydrolysis Probe Specificity

A total of 16 different bacterial species (Table S3) were used to determine the specificity of the design primer pair assay with water as no template to ensure the absence contamination using the SYBR Green assay. A 20 ng/µL gDNA from each isolate was amplified, and the mean C_T_ value was obtained. Following the primer specificity amplification, the singleplex and multiplex amplification for the probe designed was checked for its specificity, targeting sample positive control, negative control, and water for each designed probe in triplicate, and the mean CT value was obtained.

### 2.7. Broth Sample Detection Limit Evaluation

A fresh culture of *P. fluorescens* with a known concentration of 1.2 × 10^7^ CFU/mL was serially diluted 10^7^ to 10^0^ in a 0.85% saline solution (Phytotech, Beijing, China) 10-fold series. A total of 1 μL of diluted gDNA extract was taken as a template in a PCR volume mix of 20 μL, 1 μL water was used in one tube PCR mixture as a no template DNA, and 2.5 μL template DNA with each dilution series extracted was used for detection limit quantification qPCR in triplicate. Standard curves were developed by plotting CFU/mL versus the threshold cycle (C_T_) produced for the target gene. The data were analyzed using the built-in ABI 7500 software.

### 2.8. Artificial Contamination and Natural Milk Sample Experiments

A fresh culture of *P. fluorescens* with a concentration of 1.2 × 10^7^ CFU/mL estimated by plate counting was injected into the sterile UHT milk purchased from the supermarket and serially diluted about 10^7^ to 10^0^ with no incubation time. The sample was mixed and pipetted in 1 mL doses from each artificially contaminated UHT milk into a new clean 1.5 mL eppendorf tube, centrifuged at 6000 rpm for 30 min, after which the upper layer of fat was removed, the supernatant was discarded, and then the gDNA was extracted. Randomly selected 12 natural raw milk samples from the Sanxi, dairy industry in Beijing were transported to our lab at +4 °C and kept in +4 °C for 2–3 days until processed. Then, their gDNA was extracted for use and their microbial load in the milk in terms of CFU/mL was determined.

### 2.9. Data Analysis

Data analysis was conducted using qPCR soft V2.3 built-in software ABI7500 machine. For each amplification cycle denaturation step, the real-time qPCR measures the reaction of fluorescence mixture and logs the cycle number (referred to as the threshold cycle [C_T_]) where the fluorescence reaches a specific value of threshold during exponential amplification [27]. Thus, the Ct represents the amount of relevant transcript, where the target gDNA copy with the Ct value is correlated inversely. The defined Ct values were exported into Microsoft Excel for additional statistical analysis as part of the data analysis process. Student’s *t*-test was utilized for statistical significance of mean Ct value with a *p*-value < 0.05 considered statistically significant.

## 3. Result

### 3.1. Screening of Target Gene

Both the *lipA* and *aprX* genes were screened based on the multi-align sequence for the eight bacterial species of *lipA* and seven *Pseudomonas* strains of aprX fasta downloaded from the NCBI 16SrRNA partial sequence of the target and highly conserved regions with very similar sequences. Many variable regions with a total length of 362 to 442 bp were chosen with variable regions accounting for 40.62% of the whole length of the target gene each sequence. Therefore, the aligned region conserved was selected for each gene to design further primer pairs and hydrolysis probes (Figure S1).

### 3.2. Primer Pair Design and Verification

**Figure 1:**
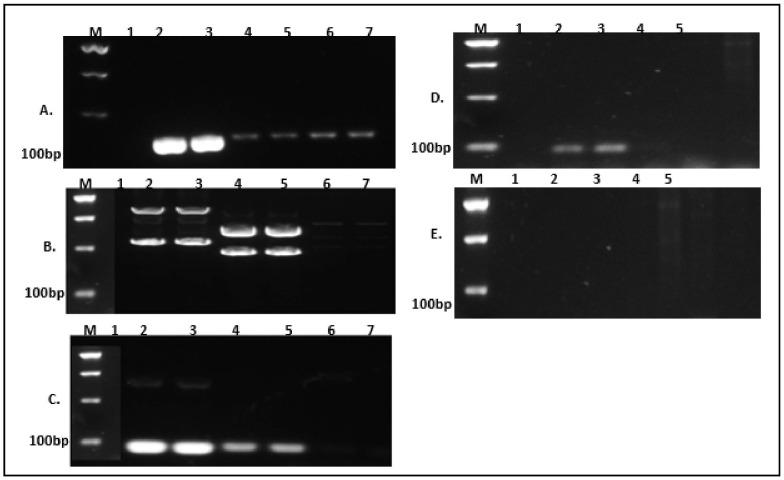
Primer pair PCR verification of the designed primer for the qPCR targeting lipA (**A**–**C**) Lanes 1 (water), 2–5 (positive), and Lanes 6,7 (negative); aprX (**D**,**E**) Lane 1 (water), Lanes 2,3 (positive), Lanes 4,5 (negative). Water: molecular-grade water, RNAse- and DNA-free.

The primer pair was designed based on the genomic context of the target region location chromosome I, description of each target using multiple alignment sequences of the target gene conserved region of the *lipA* gene and *aprX* targeting the noble strain *P. fluorescens* of the variable region primer (Figure S1) picked, respectively. The design primer pair was checked for specificity using different target strain pure culture genomic extracted DNA. Only strains with the lipA/aprX gene were amplified. In contrast, the other non-lipA/aprX gene strains and the negative control were not amplified, as shown in Figure 1C for the lipA primer, in Figure 1D for the aprX primer. At the same time, as shown in Figure 1A, there was still amplification for the negative control which means that the lipA1 primer was not specific to the target gene. However, the primer lipA2 (Figure 1B) was specific, but it still had the issue of primer dimmer while in Figure 1E, it is seen that aprX2 had no amplified products. Thus, the AGE PCR amplified result was shown in Figure 1.

### 3.3. qPCR Primer Verification

The primer pair was selected based on the amplified lower mean Ct value, where the primer with no amplification was rejected. The specificity of the selected design primer for the target gene of interest was verified using positive and negative controls as well as no template (water) in triplicate for each (Figure S2). The sensitivity of the Sybr Green qPCR assay for the target gene was evaluated using genomic DNA (gDNA) dilutions across a concentration range of 20 ng/µL to 0.0002 ng/µL. The assay demonstrated a detection limit as low as 0.0002 ng/µL, with amplification efficiency exceeding 95%.

The mean cycle threshold (Ct) values for the 16 selected target bacterial species strains are detailed in Table S3. The specificity of the qPCR primers was confirmed by testing positive strains, including the specific strain *Pseudomonas fluorescens*, using design-specific primers. For non-*Pseudomonas* lipase-producing DNA amplification products, the average Ct value was significantly higher (27.47 ± 1.25; *p* < 0.0001) compared to the target strains, which showed a mean Ct of 16.98 ± 1.76 (*p* < 0.0001). Negative controls produced Ct values > 35, while no-template controls (NTCs) yielded no detectable Ct values. The specificity of Sybr Green qPCR amplification was further verified by analyzing melting curves. All results exhibited a unique, reproducible peak melting temperature (Tm), confirming the absence of non-specific products or primer dimers.

**Figure 2:**
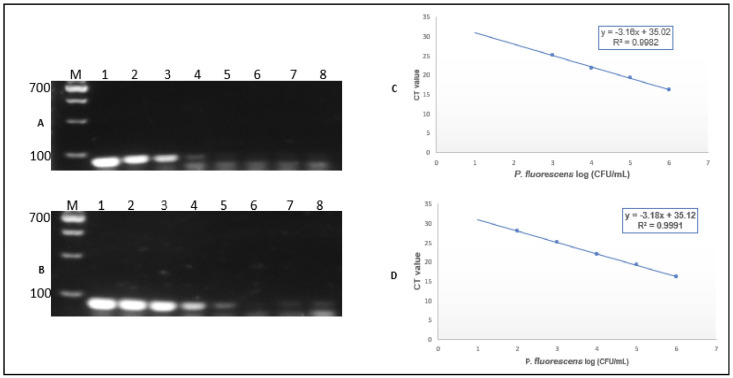
(**A**) Pure culture PCR detection, (**B**) spike milk sample PCR detection, where M is the gDNA marker, Lanes 1–8 (Lane1 1.2 × 10^6^ CFU/mL to Lane8: 1.2 × 10^−1^ CFU/mL), (**C**) pure culture establishment of a standard curve (R^2^1), and (**D**) spike milk sample established of a standard curve (R^2^2).

Detection of pure culture diluted in a 10-fold mixture, ranging from 10^6^ to 10^−1^ CFU/mL in pure broth culture and artificially contaminated UHT milk sample, was performed, where the standard curve was developed targeting the C_T_ mean value at each dilution of concentration for the noble strain *P. fluorescens* and its linear relationship between the C_T_ and log input DNA was calculated. The singleplex correlation coefficients of standard curves based on pure culture cells and the milk sample were R^2^1 = 0.9982 (slope = 3.16) and R^2^2 = 0.9991 (slope = 3.18) (Figure S3), and the minimum levels of detection were >10^4^ and >10^3^ in PCR amplification and >10^3^ and >10^2^ in real time, respectively, with the concentration series of Lanes 1–8 (lane 1: 1.2 × 10^6^ to lane 8: 1.2 × 10^−1^ CFU/mL) (Figure 2). The microbial load detection of the natural milk sample was calculated as 1.2 × 10^1^ CFU/mL, which was in the acceptable range for the sterile milk sample microbial quality, showing the detection presence of the enzyme protease and lipase in all the natural raw milk samples.

### 3.4. Taqman Probe-Based Quantification

Based on the endpoint PCR and SybrGreen primer pair verification findings, we came up with a design for a hydrolysis probe for the selected best primer targeting our gene of interest for *lipA* and *aprX* genes, which is aimed to amplify simultaneous detection in a single tube reaction using multiplex qPCR for the two target genes. For the verification of the hydrolysis probe assay fluorescence-based measurements, we used a reporter dye at 5′end fluorophores attached (FAM, VIC, Cy5) and a quencher at 3′ end attached (BHQ1, BHQ2, TAMRA, and MGB) [28] in (Table 2).

The optimum gradient temperature for quantification of the target gene based on the designed primer pair and hydrolysis probe annealing temperature for the qPCR ranges from 62 °C to 58.5 °C with an increment of −0.5 °C, and it was set to be 60 °C. Then, we quantified each representative positive, negative, and water with a probe concentration (150 nM).

The *lipA* and *aprX* genes are commonly used as genetic markers to detect spoilage bacteria in milk and dairy products, particularly those belonging to the *Pseudomonas* genus [29,30]. These bacteria are the major concerns in milk spoilage since they release heat-resistant extracellular hydrolysis enzymes lipases and proteases that can spoil milk and dairy products even after pasteurization [31]. Here, in Figure S4, a comparison was presented of the efficiency of the *lipA* and *aprX* genes in detecting spoilage microbes in raw milk with a mean Ct of each amplification based on the target probe design, where Target 1 shown in Figure S4A,B was for *lipA,* Target 1A was lipA6 with low mean Ct, Target 2 in Figure S4C,D was for *aprX,* Target 2C was aprX1 with lower Ct. These values were taken as quantification primers and probes chosen for the intended experiment based on the criteria of the lower mean Ct value and good amplification efficiency. The executed mean Ct value of each target was compared, and the lowest Ct value was considered for qPCR quantification, for use as a probe of choice for the experiment based on one positive control pseudomonas, two positive non-pseudomonas, negative control, and no template for each gene of interest in triplicate. Each sample’s result was interpreted in terms of a singleplex with target and sample-based quantifications.

Then, finally, we decided to compare and offer justification for the multiplex qPCR findings with two positive pseudomonas, two positive non-pseudomonas, negative control milk DNA, and no template in a single PCR tube in triplicate to make sure there is no interference and cross-reactivity compared to the singleplex qPCR result. Multiplex qPCR appeared a promising method for the simultaneous detection of the two target genes and was effective in amplifying each target gene in a single tube. Therefore, as per the findings, multiplex qPCR was more sensitive, cost-effective, and time-saving to detect and quantify than a singleplex qPCR.

**Figure 3:**
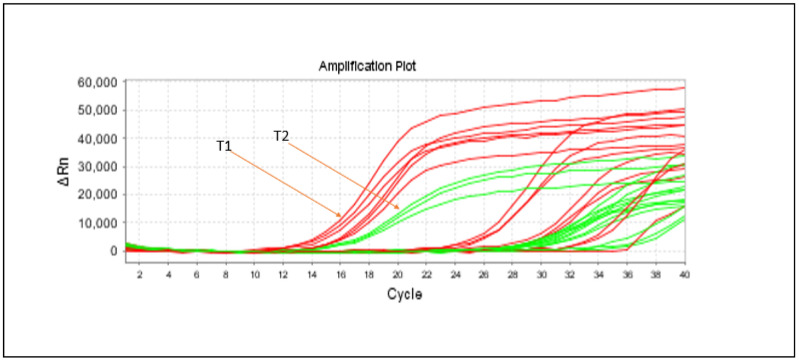
Development of simultaneous detection using multiplex qPCR amplification for the two target genes in a single tube PCR, targeting *Pseudomonas fluorescens* (T1: Target1 (*lipA*), T2: Target2 (*aprX*))**.**

The sensitivity comparison between the two target probes reveals that Target 1 (*lipA*) exhibits greater sensitivity than Target 2 (*aprX*), as demonstrated in Figure 3; however, this finding contrasts with conclusions from some researchers [32,33], who reported that *aprX* shows higher sensitivity for microbial detection in raw milk compared to *lipA* in their studies. This discrepancy highlights potential context-dependent variability in assay performance, possibly influenced by sample type or experimental conditions.

**Figure 4:**
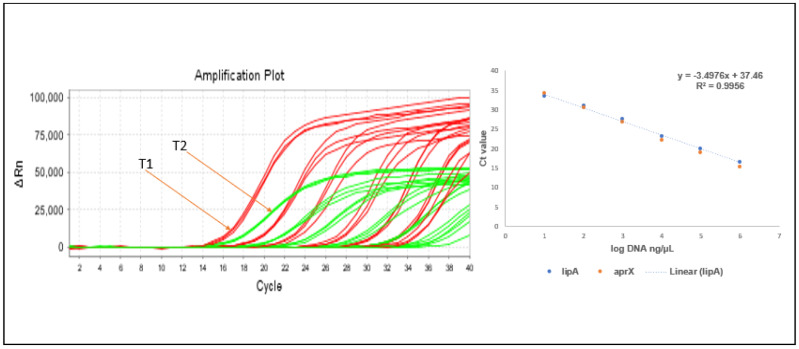
The simultaneous detection limit of multiplex qPCR for Target 1 (*lipA*) and Target 2 (*aprX*) gene, with an average Ct value and the logarithm of the concentration of gDNA of target genes (T1: Target 1 (*lipA*), T2: Target 2 (*aprX*)).

The sensitivity detection of singleplex qPCR for the target template DNA was performed, with the DNA concentration increasing 10-fold in a dilution series from 20 ng/µL to 0.0002 ng/µL. The coefficient of regression value R^2^1 for Targets 1 (*lipA*) and 2 (*aprX*) (0.9908) was on a slope (−3.2591) and intercepted with (36), where the percent of efficiency (%E) of the single plex qPCR reached the level of 95.80%. The sensitivity detection of multiplex regression value R^2^2 for Targets 1,2 (0.9956) was on a slope (−3.4974) and intercepted with (37). The percent of efficiency (%E) reached the level of 102.69%, and both could detect up to 0.0002 ng/µL. Amplification efficiency (E) of the qPCR was calculated as E = 10^(−1/slope)^ and the percent of efficiency (%E) was calculated as %E= (E − 1) × 100% [34]. Therefore, here, we confirm that there is no cross-reaction sensitivity and no interference for the multiplex qPCR, though the two target probes combine to quantify the target gene of interest in a single tube PCR as we see in Figure 4. The duplex amplification curve ΔRn of *aprX* is higher than that of lipA. As the concentration of the DNA decreases, the fluorescence signal decreases in both cases.

## 4. Discussion

Phenotypic and biochemical approaches have been the standard for identifying milk microbial composition [35]. These procedures are labor-intensive and need a technician to spend a lot of time handling them during setup and continuous quality control [36]. To enable more accurate strain-to-species identification, genotype-based identification techniques extend beyond the issue of variable phenotypes [37]. Recently, methods for identifying microbials based on their molecular characteristics have been devised, targeting the specific gene. However, the deficiency and mutation of some gene factors in strains can lead to a false positive and even present a risk of food spoilage [38]. With the advancement of sequencing technology and bioinformatics, a huge number of microbial genome sequences have been described and published [39]. As a result, many researchers have concentrated on investigating and screening novel specific target markers that may be able to substitute some target genes with low specificity [38,40].

In this research study, we develop a real-time multiplex qPCR method to detect hydrolytic enzyme-producing strains targeting the noble strain *P. fluorescens* in raw milk. The technique is designed to detect the two-target enzyme coding lipase (*lipA*) and alkaline metalloprotease (*aprX*) *genes* which are important in milk and dairy products because they encode enzymes that can lead to spoilage [41]. Lipase, a lipolytic enzyme, can break down milk fat (triglycerides), producing free fatty acids and glycerol that contribute to off-flavors and rancidity [42]. *AprX*, an alkaline metalloprotease, can degrade various proteins, including casein in milk, leading to bitter flavors and changes in texture [43]. These enzymes are often produced by Psychrotrophic bacteria, which can survive and grow in refrigerated conditions, thus contributing to the spoilage of dairy products with long shelf lives [44]. In milk bacteria, especially strains of *Pseudomonas*, the *lipA* gene plays a significant part in the milk spoilage potential of refrigerated by-products since the lipolytic activity of these enzymes [44,45]. The triacylglycerol lipase gene (*lipA*) is the preferred target for primer pair and hydrolysis probe design, used for PCR and real-time PCR. While the *aprX* gene encodes alkaline metalloproteases, it is a key marker primarily associated with *Pseudomonas fluorescens* which are commonly found in milk and dairy products [43]. These bacteria thrive at refrigeration temperatures and produce heat-stable enzymes that can degrade proteins and lipids, leading to bitterness and coagulation, causing gelation, off-flavors, and texture defects in dairy products [29].

The designed primer pair specificity is confirmed by evaluating different strains of bacteria gDNA extracted and amplifying them using end-point PCR and qPCR applications. All *Pseudomonas* and non-*Pseudomonas* enzyme producing strains with both target genes have positive PCR amplification products, while all non-enzyme content strains provide negative amplification for the agarose gel electrophoresis PCR. The positive strains quantified in this research method, including the *P. fluorescens* strain, show a constant peak Tm of 86 °C with a mean Ct value of 16.98 ± 1.76, 27.47 ± 1.25 for enzyme-producing and no Ct for the negative control, respectively, with a standard curve showing a strict inverse correlation in broth and artificially contaminated UHT, where the sensitivity reaches >10^3^ and >10^2^ CFU/mL, respectively, where the sensitivity of the natural milk sample is deemed in the acceptable range of 1.2 × 10^1^ CFU/mL, and with a gDNA sensitivity up to 0.0002 ng/µL. These are the same findings as previously reported by Liang et al., a study performed on detection and quantification of Bacillus cereus and its spores in raw milk [46,47].

The TaqMan probe method is applied in this research where each quencher’s function is to absorb the fluorescence emitted by the reporter dye, preventing it from being detected when the probe is intact and thus suppressing the fluorescent signal [48,49]. This results in a low background signal during the initial stages of a PCR, enhancing the clarity of the data as the amplification progresses for comparison of gene detection sensitivity of the *lipA* and *aprX.* Primer and hydrolysis probes are designed for each target base real-time qPCR with a mean Ct value for the selected strains of both pseudomonas and non-pseudomonas enzyme producing strains and negative control quantification with consistent values of 16, 28, and 36, respectively, and no Ct for the no template sample. This shows that Taqman probe detection is more sensitive than the Sybr green compared to each Ct value. Comparing the specificity and efficiency of each mean Ct value for each singleplex with the multiplex real-time qPCR for Target 1 and Target 2, their correlation standard curve values show R^2^1: 0.9908 and R^2^2: 0.9956 with a significance efficiency > 95%, indicating that there was no interference for combining the two targets in a single PCR tube. Thus, multiplex qPCR is more specific, sensitive, time-saving, and cost-effective in determining the detection of the target spoilage bacteria in milk and dairy products. This method was affirmed as a promising approach by Wu et al. [50] and the comprehensive approach in that study for monitoring spoilage potential in milk. Another method developed by Maier et al. [51] describes well the powerful use of multiplex quantification, thereby furthering its future application to predict spoilage risk and shelf life of raw milk at an early stage.

## 5. Conclusions

In general, with the advancement in detection technology, conventional PCR using gel electrophoresis to separate the amplified product and then hybridizing it with a probe are standard PCR procedures. Nowadays, the detection technology of milk microbes is becoming accurate, quicker, and more convenient; thus, qPCR quantification assays are taking the place of these laborious methods to identify pathogen and spoilage microorganisms in milk samples; the real-time multiplex qPCR method outlined in this research study is straightforward, accurate, specific, and helps in early detection, which could help improve and milk product quality.

The limitation of this study is the use of a limited number of bacterial strains. While the primary objective is to develop a multiplex qPCR assay for simultaneous detection of enzyme coding gene markers responsible for milk spoilage, the enzymatic activity of psychrotrophic bacteria critical to understanding spoilage dynamics is not analyzed. The multiplex qPCR method shows promise as a rapid, cost-effective tool for detecting spoilage-related psychrotrophic bacteria in raw milk. By enabling simultaneous identification of target enzyme coding gene markers, this approach could streamline monitoring of spoilage potential, reduce processing time, and enhance quality control in the dairy industry. Future studies should integrate enzymatic activity assessments to further refine the correlation between enzyme-encoding genes and functional spoilage outcomes for improving milk quality and monitoring.

## Figures and Tables

**Table 1:** List of selected bacterial strains for the experiment. (+ve: enzyme hydrolysis positive, −ve: enzyme hydrolysis negative).

No	Bacteria	NCBI Acc. No	Lipase/Protease (+Ve/−Ve)
1	*Enterococcus hirae*	NR_037082	−ve
2	*Acinetobacter indicus*	NZ_CP045198	+ve
3	*Pseudomonas fluorescens*	NZ_LT907842	+ve
4	*Serratia liquefacients*	NZ_MQRG01000035	+ve
5	*Pseudomonas azotoformans*	NZ_LT629702	+ve
6	*Pseudomonas paralactis*	NR_156987	+ve
7	*Exiguobacterium indicum*	NR_042347	−ve
8	*Pseudomonas putida*	NC_021505	+ve

**Table 2:** Primer pair list with their hydrolysis probe design specific to 5′ reporter and 3′ quencher attached with fluorescence signals. (NB:lipA3 and lipA6 are among the best selected primer designs from the total of 6 lipase gene primers, while aprX1 and aprX2 are also among the best selected designed primers for the alkaline metalloprotease aprX gene).

Primer	5′ to 3′	Annealed Bases	bp
lipA 3	F- ACGTGGTGATCACTTCGGTA	20	73
	R-CGAATGCAGTCGGCAAAGTG	20	
	P-FAM-CCGTCACGCAGGTCGTCGCG-TAMRA	20	
lipA 6	F-CTCAGCACTTTGCCGACTG	19	72
	R-GAACCAGGGTTTCGAGCATC	20	
	P-FAM-CCGCAAGCTGTCGCCGAACGT-MGB	21	
aprX 1	F-AACGGCAACCCGACCTATAA	20	85
	R-TGTTGCTCTCGCTCCAGTAA	20	
	P-Cy5-CGCAACCTATGGACAGGACACGCG-BHQ2	24	
aprX 2	F-CGGACCTGAACAACTATGGC	20	109
	R-TTATAGGTCGGGTTGCCGTT	20	
	P-Cy5-TCGGCCACACCCTGGGCCTG-BHQ1	20	

## Data Availability

The original contributions presented in this study are included in the article/Supplementary Materials. Further inquiries can be directed to the corresponding author.

## Supplementary Materials

The following supporting information can be downloaded at: https://www.mdpi.com/article/10.3390/foods14111975/s1, Table S1. List of isolate strains amplified using 16S gene sequence with their extracellular hydrolysis profile and accession number. Table S2. List of primer pair designs for the intended qPCR amplification of the target genes lipA and aprX with their annealed basses and bp size. Table S3. Primer specificity and cross-reactivity verification. Figure S1. (A) Multiple align sequences of the target gene fasta 16s rRNa partial sequence of selected strains for both lipA & aprX gene, (B) Central Chromosome I location, and primer pair selection. Figure S2. A.verification of lipA primer B. Verification of aprX primer C. Specificity of selected working primer verification D. Sensitivity of the primer pair with gDNA concentration 20 ng/µL to 0.0002 ng/µL. Figure S3. The coefficient of efficiency for each singleplex target gene of lipA (A) and aprX (B). Figure S4. Each amplification result shows the best probe selection among each design for each target gene Ct value lipA (A: probe 6, B: probe 3) and aprX (C: probe 7, D: probe 8).
