# Supplementary material for: Development of Multiplex qPCR Method for Accurate Detection of Enzyme-Producing Psychrotrophic Bacteria
Source: Foods. 2025 Jun 3;14(11):1975. doi: 10.3390/foods14111975 (PMC12154344; doi:10.3390/foods14111975)
Supplement: Supplementary file 1 [file foods-14-01975-s001.zip › Supplementary Table with caption.pdf]

List of Tables

Table S1. List of isolate strains amplified using 16S gene sequence with their extracellular hydrolysis profile and accession number.

No	Bacteria	Accession number	Extracellular hydrolysis test
1	Pseudomonas rhodesiae	MH671644.1	+
2	Pantoea ananatis	OR481731.1	+
3	Pseudomonas lactis	OR272042.1	+
4	Mammaliicoccus sciuri	PP737879.1	+
5	Staphylococcus sciuri	MN535879.1	+
6	Pseudomonas putida	KU512626.1	+
7	Exiguobacterium sibiricum	MH712973.1	-
8	Psychrobacter maritimus	KP236243.1	+
9	Acinetobacter lwoffii	PP467476.1	+
10	Enterobacter cloacae	OR516804.1	-
11	Acinetobacter beijerinckii	OQ983648.1	+
12	Pseudomonas poae	OK037030.1	+
13	Pseudomonas lundensis	MN758769.1	+
14	Psychrobacter maritimus	MT131386.1	+
15	Pseudomonas veronii	KU323913.1	+
16	Lactococcus garvieae	OR502221.1	-
17	Chryseobacterium oleae	MN513215.1	-
18	Pseudomonas putida	MT626824.1	+
19	Pseudomonas jessenii	JX426063.1	+
20	Pseudomonas koreensis	MT362708.1	+
21	Rouxiella badensis	ON626777.1	-
22	Pseudomonas paralactis	MT373668.1	+
23	Acinetobacter johnsonii	MN733098.1	+

24	Enterococcus lactis	OR502297.1	-
25	Enterococcus faecalis	OR513080.1	-
26	Acinetobacter albensis	MN758790.1	+
27	Sanguibacter inulinus	MT032322.1	-
28	Enterococcus hirae	KX553842.1	-
29	Pseudomonas fluorescens	KR267334.1	+
30	Serratia liquefaciens	NZ_CP048784.1	+
31	Pseudomonas azotoformans	LC159299.1	+
32	Pseudomonas gessardii	MT626825.1	+
33	Pseudomonas marginalis	HE586393.1	+

Table S2. List of primer pair designs for the intended qPCR amplification of the target genes lipA and aprX with their annealed basses and bp size

Primer list	5' to 3'	Annealed bases	bp
**lipA 1**	F-CAGTGTGACGGCATGGT	17	94
	R-ACTGGTTGACGATATCGAAGTG	22	
**lipA 2**	F-GGAGAAGGGCCAGTGTGA	18	110
	R-CCAGGGACTGGTTGACGATA	20	
**lipA 3**	F- ACGTGGTGATCACTTCGGTA	20	73
	R-CGAATGCAGTCGGCAAAGTG	20	
**lipA 4**	F-GAACCTGGGTGAGTGCTTCT	20	193
	R-GCGGTCTACCGAGGTGATTA	20	
**lipA 5**	F-ACCCTGGTTCCTGACTATCG	20	165
	R-GAATTTCTGCAGCAGGGTCA	20	
**lipA6**	F-CTCAGCACTTTGCCGACTG	19	72
	R-GAACCAGGGTTTCGAGCATC	20	
aprX 1	F-AACGGCAACCCGACCTATAA	20	85
	R-TGTTGCTCTCGCTCCAGTAA	20	
aprX 2	F-CGGACCTGAACAACTATGGC	20	109
	R-TTATAGGTCGGGTTGCCGTT	20	
aprX 3	F-ACGCGTGGTTATAGCCTCAT	20	66
	R-GCCTTTGCTGAAGTTCTGGT	20	

Table S3. Primer specificity and cross-reactivity verification

S/no	Strain name	Accession number	Lipase	Mean C_T value
1	Sanguibacter inulinus	MT032322.1	-	No Ct
2	*Exiguobacterium sibiricum*	MH712973.1	-	No Ct
3	*Pseudomonas fluorescens*	KR267334.1	+	15.43
4	*Enterobacter cloacae*	OR516804.1	-	No Ct
5	Sanguibacter inulinus	MT032322.1	-	No Ct
6	*Lactococcus garvieae*	OR502221.1	-	No Ct
7	*Pseudomonas azotoformans*	LC159299.1	+	16.19
8	*Sanguibacter inulinus*	MT032322.1	-	No Ct
9	*Enterococcus hirae*	KX553842.1	-	No Ct
10	*Acinetobacter lwoffii*	PP467476.1	+	27.45
11	*Lactococcus garvieae*	OR502221.1	-	No Ct
12	*Enterococcus faecalis*	OR513080.1	-	No Ct
13	*Enterococcus hirae*	KX553842.1	-	No Ct
14	*Pseudomonas lactis*	OR272042.1	+	16.22
15	*Enterobacter cloacae*	OR516804.1	-	No Ct
16	*Serratia liquefacients*	NZ_MQRG01000035	+	28.34
