# Supplementary material for: Development of Multiplex qPCR Method for Accurate Detection of Enzyme-Producing Psychrotrophic Bacteria
Source: Foods. 2025 Jun 3;14(11):1975. doi: 10.3390/foods14111975 (PMC12154344; doi:10.3390/foods14111975)
Supplement: Supplementary file 1 [file foods-14-01975-s001.zip › Supplementary Figure with caption.pdf]

Supplementary figures only

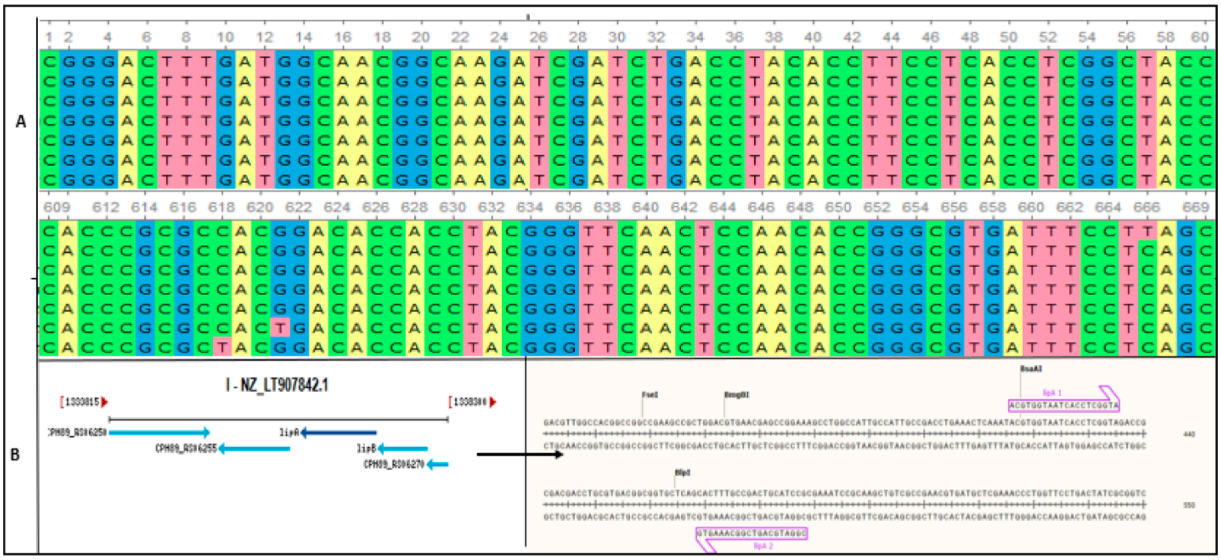

Figure S1. Illustration of conserved region selection and primer design

(A) Multiple align sequences of the target gene fasta 16s rRNa partial sequence of selected strains

(B) Central Chromosome I location, and primer pair selection

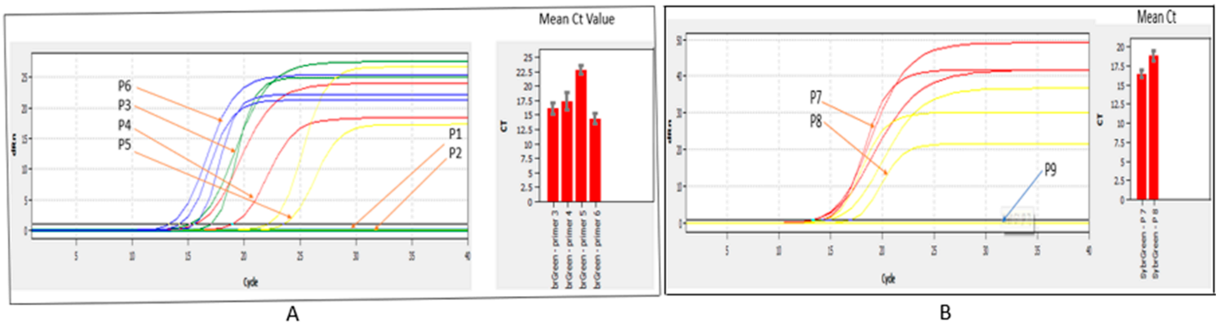

A B

Well		Sample name	Sample type	Gene	Ct	Mean Ct
G2		P6	NTC		No Ct	
G3		P6	NTC		No Ct	
G4		P6	NTC		No Ct	
G5		P6	Negative contro		No Ct	
G6		P6	Negative contro		No Ct	
G7		P6	Negative contro		No Ct	
G8		P6	Positive control		16.09	16.85
G9		P6	Positive control		17.37	16.85
G10		P6	Positive control		17.1	16.85

C

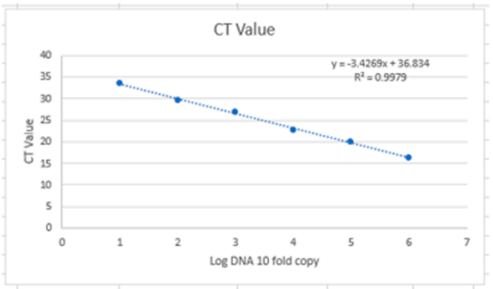

D

Figure S2. A.verification of lipA primer B. Verification of aprX primer C. Specificity of selected working primer verification D. Sensitivity of the primer pair with gDNA concentration 20ng/µL to 0.0002ng/µl

A

B

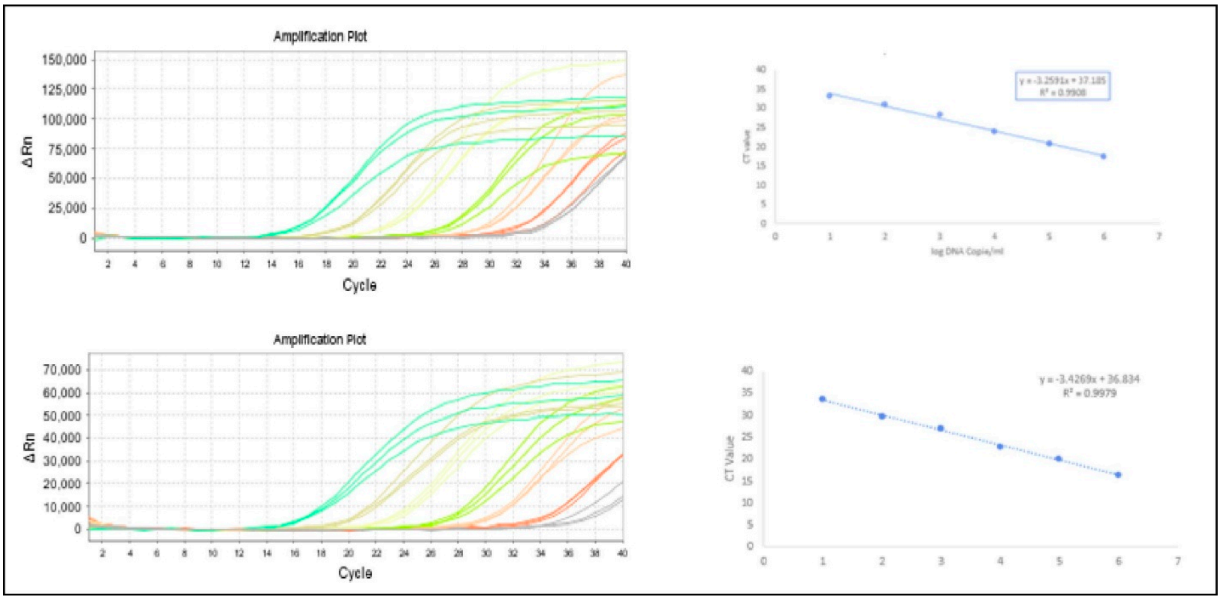

Figure S3. The coefficient of efficiency for each singleplex target gene of lipA (A) and aprX (B)

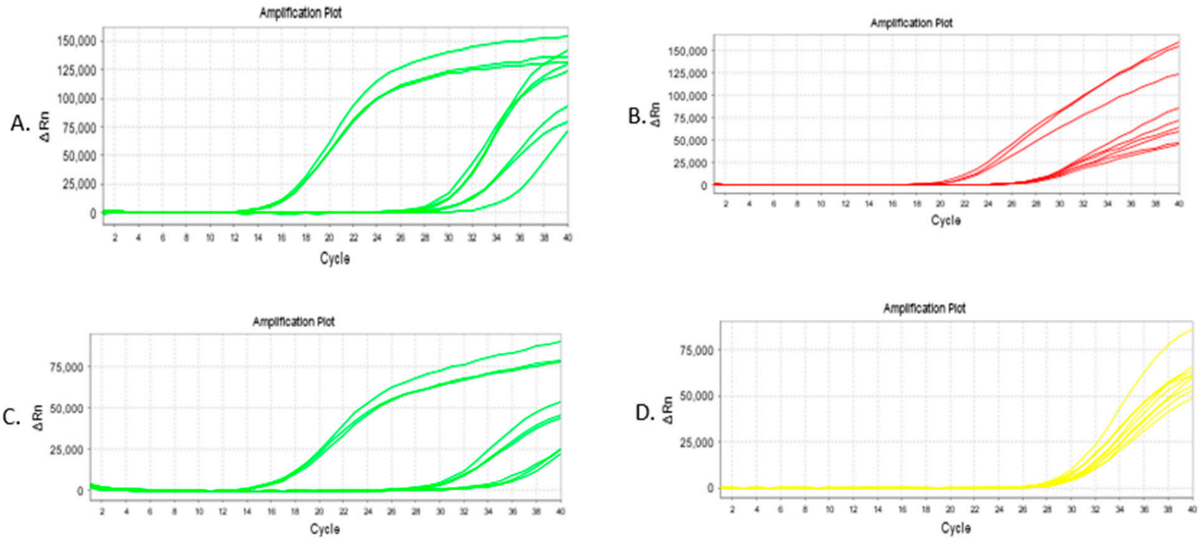

Figure S4. Each amplification result shows the best probe selection among each design for each target gene Ct value lipA (A:probe 6, B:probe 3) and aprX (C:probe 7, D:probe 8)
